# Interspecies Hybridisation and Genome Chimerisation in *Saccharomyces*: Combining of Gene Pools of Species and Its Biotechnological Perspectives

**DOI:** 10.3389/fmicb.2018.03071

**Published:** 2018-12-11

**Authors:** Matthias Sipiczki

**Affiliations:** Department of Genetics and Applied Microbiology, University of Debrecen, Debrecen, Hungary

**Keywords:** interspecies hybridisation, sterility, alloploid, meiosis, genome chimerisation, strain improvement, fermentation, yeast

## Abstract

Over the last one and a half decade, interspecies hybridisation has gained continuously increasing attention as a breeding technique suitable for transferring of genetic information between *Saccharomyces* species and mixing of their gene pools without genetic engineering. The hybrids frequently show positive transgressive phenotypes. Segregation of the hybrid genome results in mosaic (chimeric) strains that can outperform both the parents and the hybrids or exhibit novel positive phenotypic properties. Mitotic segregation can take place during the vegetative propagation of the sterile allodiploid hybrid cells. Meiotic segregation becomes possible after genome duplication (tetraploidisation) if it is followed by break-down of sterility. The allotetraploid cells are seemingly fertile because they form viable spores. But because of the autodiploidisation of the meiosis, sterile allodiploid spores are produced and thus the hybrid genome does not segregate (the second sterility barrier). However, malsegregation of *MAT*-carrying chromosomes in one of the subgenomes during allotetraploid meiosis (loss of *MAT* heterozygosity) results in fertile alloaneuploid spores. The breakdown of (the second) sterility barrier is followed by the loss of additional chromosomes in rapid succession and recombination between the subgenomes. The process (genome autoreduction in meiosis or GARMe) chimerises the genome and generates strains with chimeric (mosaic) genomes composed of various combinations of the genes of the parental strains. Since one of the subgenomes is preferentially reduced, the outcome is usually a strain having an (almost) complete genome from one parent and only a few genes or mosaics from the genome of the other parent. The fertility of the spores produced during GARMe provides possibilities also for introgressive backcrossing with one or the other parental strain, but genome chimerisation and gene transfer through series of backcrosses always with the same parent is likely to be less efficient than through meiotic or mitotic genome autoreduction. Hybridisation and the evolution of the hybrid genome (resizing and chimerisation) have been exploited in the improvement of industrial strains and applied to the breeding of new strains for specific purposes. Lists of successful projects are shown and certain major trends are discussed.

## Introduction

Strains of *Saccharomyces cerevisiae*, the major yeast used in fermentation technologies (Tuite and Oliver, 1991) show high genetic and phenotypic diversity. The favorable properties of the different strains can be brought together by hybridisation. The hybrids not only show transgressive phenotypes but also produce mitotic and meiotic segregants of diverse combinations of the traits of the hybridizing partners. Strains of the other species of the genus can have additional favorable properties but their combination with those of the *S. cerevisiae* strains is hampered by the postzygotic reproductive isolation of the species manifested as hybrid sterility. The interspecies hybrids of the *Saccharomyces* species are viable but either do not produce gametes (ascospores) or if they do so, the viability of the gametes is extremely low. This sterility barrier keeps the species biologically isolated but the isolation is not absolute. The hybrid genomes can change and certain types of changes make the barrier permeable. In a previous review a model was proposed to integrate these postzygotic events into a coherent system based on what was then known (Sipiczki, 2008). According to the model, the hybrid genome undergoes a gradual size reduction by losing chromosomes, either in the course of vegetative propagation of the allodiploid hybrid cells or during allotetraploid meiosis which takes place upon spontaneous genome duplication. Concomitantly with size reduction the subgenomes can interact and recombine. The “stabilized” outcomes of these processes are recombinant haploids and aneuploids, in fact strains with chimeric (mosaic) genomes. Hybridisation and postzygotic genome chimerisation can be observed in the laboratory but can take place also in natural habitats as demonstrated by the occurrence of chimerised genomes in strains isolated from yeast communities fermenting beverages. Over the past 10 years, considerable progress has been made in the investigation of hybrid sterility, the breakdown of the sterility barrier, and the mechanisms underlying the postzygotic reduction and chimerisation of the hybrid genome. These processes and their exploitation in the improvement of industrial *Saccharomyces* strains, as a non-GMO alternative of targeted genetic manipulation, are the subjects of this review. A review of this length cannot be comprehensive and thus it will not cover the hybrid species, the natural “hybrid strains” and the evolutionary aspects of hybridisation. The reader interested in the developments in these fields can consult review papers (e.g., Sipiczki, 2008; Louis, 2011; Albertin and Marullo, 2012; Morales and Dujon, 2012; Dujon and Louis, 2017; Krogerus et al., 2017a; Bisson, 2017; Gibson et al., 2017; Guillamón and Barrio, 2017; Lopandic, 2018) published elsewhere. Given that certain genetic terms are often inconsistently used in the literature, a section will address terminological issues.

## Taxonomy of *Saccharomyces* (*Saccharomyces* sensu stricto)

The taxonomy of *Saccharomyces* changed many times in the history of the genus. van der Walt (1970) separated the highly fermenting species from the rest of the genus and proposed the name *Saccharomyces* sensu stricto complex for them. Since then the species not included in this group (sensu lato) were transferred to other genera, so the name “sensu stricto complex” has become obsolete. Yeast taxonomy currently accepts 7 “natural,” “clean” or “single-genome-based” [*S. arboricolus* (*S. arboricola*), *S. cariocanus, S. cerevisiae*, *S. kudriavzevii*, *S. mikatae*, *S. paradoxus*, and *S. uvarum* (*S. bayanus* var. *uvarum*)] and 2 “hybrid” [*S. pastorianus/carlsbergensis* and *S. bayanus* (*S. bayanus* var. *bayanus*)] *Saccharomyces* species (Vaughan-Martini and Martini, 2011). Since 2011 two new *Saccharomyces* species *S. eubayanus* and *S. jurei* were described. However, this classification is in contradiction with the results of whole-genome sequencing. Whole-genome analysis has shown that *Saccharomyces cariocanus* should be considered to be more a *S. paradoxus* variant as this species is reproductively isolated from *Saccharomyces paradoxus* by four translocations but not by sequence (reviewed in Borneman and Pretorius, 2015; Dujon and Louis, 2017; Nguyen and Boekhout, 2017). But it does not fit with mtDNA gene order, which is considered as a species-specific feature. The *S. cariocanus* mtDNA is not syntenic to that of *S. paradoxus* and therefore *S. cariocanus* should be designated as a separate species (Sulo et al., 2017).

The hybrid species (*S. bayanus*, and *S. pastorianus/ carlsbergensis*) occurring almost exclusively in beer fermentation are highly heterogeneous in genome structure and the proportion of the genomic mosaics originating from the assumed parental species (e.g., Nguyen and Gaillardin, 2005; Rainieri et al., 2006; Dunn and Sherlock, 2008; Nakao et al., 2009; Libkind et al., 2011; Nguyen et al., 2011; Walther et al., 2014; Pérez-Través et al., 2014a; Van den Broek et al., 2015). In addition to these hybrid species, many other *Saccharomyces* strains have been identified mainly among wine yeasts that contained mosaics of foreign origin in their genomes. These strains usually referred to as “hybrids” are not considered distinct taxonomic entities (for reviews, see Sipiczki, 2008; Albertin and Marullo, 2012; Morales and Dujon, 2012; Dujon and Louis, 2017; Lopandic, 2018). There is no consensus about where to draw the line between the hybrid species and the interspecies hybrids. Both groups have diverse chimeric genomes.

There is considerable confusion also around the taxonomic identity of *S. bayanus* and *S. uvarum*. Both species have been described in the 19th century but because of their proposed merger under the name *S. bayanus* some three decades ago (Vaughan-Martini and Kurtzman, 1985), the name *S. uvarum* has become sparsely used in the literature until recently. However, the molecular genetic and genomic analyses clearly separated two groups, a clean and a hybrid group, among the strains that had been assigned to *S. bayanus* over the last 30 years and led to the reinstatement of the species *S. uvarum* for the clean group (Nguyen and Gaillardin, 2005; Nguyen et al., 2011). As most *S. bayanus* strains isolated from substrates not related to beer production have turned out in molecular tests to be conspecific with the type strain of *S. uvarum*, the name *S. bayanus* will be restricted only to brewery yeasts in this review.

All discrepancies in fungal taxonomy come from the three species concepts that have been applied to species delimitation: the classical morphology/physiology-based, the biological and the evolutionary/phylogenetic species concepts. The classical concept is difficult to apply generally in *Saccharomyces* taxonomy because of the strain variation in the utilization of different substrates and the absence of distinctive morphological properties. The biological species concept, which defines a species as a group of actually or potentially inbreeding natural population reproductively isolated from other such groups (Mayr, 1940), was introduced to *Saccharomyces* taxonomy by Naumov (1987) and successfully applied to the description of novel species and to the determination of the taxonomic affiliation of novel isolates. This approach is based on the observation that the hybrids of the *Saccharomyces* strains are viable, but only hybrids of conspecific strains can produce viable spores at frequencies higher than 1%. However, this method is impractical in large-scale screening and routine testing of taxonomic affiliation of larger numbers of isolates. The phylogenetic concept defines a species as a monophyletic group of organisms sharing molecular characters that derive from a common ancestor (Moore et al., 2011). The phylogenetic relationships of yeast strains can be examined by comparing the sequences of their evolutionary conserved genes or non-coding chromosomal segments such as the D1/D2 domains of the genes encoding the large-subunit (LSU) rRNA molecules and the internal transcribed segments (ITS), two components of the repeats of the rDNA arrays. A recent study of 9,000 yeast isolates of the CBS collection (Vu et al., 2016) found that strains differing by more than 0.49% in their D1/D2 domains and by more than 1.59% in their ITS segments are usually not conspecific. The “natural” *Saccharomyces* species can be separated with these criteria. The “hybrid species” *S. pastorianus/carlsbergensis* and *S. bayanus* are difficult to distinguish from the “natural species” because certain their strains show no sexual activity (untestable for biological isolation) and/or have rDNA sequences of different “natural” species in heterozygous constitution (e.g., Nguyen and Gaillardin, 2005; Rainieri et al., 2006; Nguyen and Boekhout, 2017). Nevertheless their type strains sporulate and it is possible to mate them e.g., to *S. cerevisiae* (Špírek et al., 2014). The pan-genome analysis (e.g., Tettelin et al., 2005; Dunn et al., 2012) of large numbers of strains of all species of the genus may shed new light on the problem of species boundaries.

## Terminological Difficulties

### Authenticity of Species

The widespread view is that the *Saccharomyces* species have the same number (16) of mostly syntenic nuclear chromosomes (e.g., Fischer et al., 2000; Kellis et al., 2003; Scannell et al., 2011; Dunn et al., 2013; Liti et al., 2013; Baker et al., 2015). This view based on the comparison of a limited number of structurally assembled genome sequences is at odds with the high intraspecies diversity of karyotypes and genome structures observed in *S. cerevisiae* and *S. uvarum* isolates (e.g., Johnston and Mortimer, 1986; Bakalinsky and Snow, 1990; Yamamoto et al., 1991; Adams et al., 1992; Bidenne et al., 1992; Kishimoto and Goto, 1995; Miklos et al., 1997; Sipiczki, 2002; Csoma et al., 2010; Leducq et al., 2016; Nguyen and Boekhout, 2017; Albertin et al., 2018). If this diversity is not taken into account, the interpretation of the results of the hybridisation experiments can easily lead to disputable conclusions. Perhaps it is more accurate to conceive each species as a population of diverse strains that share certain species-specific attributes but may show diversity in other properties and also in genome structure. For example, it does not seem substantiated to declare a sequenced genome “the *S. cerevisiae* genome” and another sequenced genome “the *S. uvarum* genome” and claim that these two species differ in specific translocations or inversions that occurred a certain time ago. This claim is correct if only two strains are compared. However, other strains of these species show more or different structural genome differences including mosaics of foreign origin attributable to more recent events.

### Hybrid – Mosaic – Genetically Admixed – Chimera – Evolved Hybrid

Further confusion can be caused by the presence of genes of different species in the genome. Genomes containing foreign genes were found in many strains isolated from yeast populations of fermenting beverages or constructed in laboratories. Many isolates identified as *S. cerevisiae* (for reviews, see e.g., Sipiczki, 2008; Morales and Dujon, 2012; Lopandic, 2018) or *S. uvarum* (e.g., Almeida et al., 2014; Albertin et al., 2018) turned out to have genes of foreign origin (alien genes) in their genomes. It can be assumed that, when subjected to molecular testing, many more wine and ale strains of these species will turn out to have genomic mosaics acquired from other species. The question arises as to whether the presence of such alien genes is characteristic of all yeasts fermenting alcoholic beverages as a consequence of “domestication.” These strains pose a terminological problem. They can be considered conspecific with one or the other species or can be regarded as interspecies hybrids. The latter term has become widely used even for strains in which only one or two foreign genes were detected. The application of terms like “natural *S. cerevisiae* × *S. kudriavzevii* hybrid” or “natural *S. cerevisiae* × *S. kudriavzevii* × *S. uvarum* hybrid” to such strains can be confusing because it conceals the exact genetic identity of the strains and may lead to the erroneous assumption that these strains have no taxonomic affinities. Certain authors (e.g., Groth et al., 1999; Belloch et al., 2009; Sipiczki, 2011) tried to resolve the confusion by applying the term “chimera” or “genomic chimera” (used in other groups of organisms; e.g., Jansen et al., 2011; Watanabe et al., 2012; Pryszcz et al., 2014) to *Saccharomyces* strains having mosaics of different origin in their genomes. Unfortunately, this has not become a widely used practice. Nevertheless, for the sake of ambiguity, the term chimera (chimeric) will be used in this review to distinguish strains having mosaic genome structures from “true” hybrids. Natural true hybrids having complete genomes of two species were rarely found (e.g., Masneuf et al., 1998; Naumov et al., 2000; Borneman et al., 2012). A good alternative for “chimeric” and “mosaic” can be “admixed” (e.g., Tilakaratna and Bensasson, 2017).

In the case of the hybrids constructed in laboratories, the terminological diversity is not smaller. The most frequently used names (synonyms) are synthetic, artificial, constructed, laboratory-constructed, newly formed, de novo and experimental hybrids. Derivatives of the hybrids which usually have reduced and chimeric genomes are often called “evolved hybrids” instead of the more accurate “segregants.”

### HTG – Introgression – Genetic Admixture – Genome Autoreduction

Formally, the foreign genes/mosaics are horizontally transferred segments of the genome(s) of the donor species to the chimeric (“hybrid”) strains of the acceptor species. Their origin can be traced back by searching databases for similar sequences, but the mechanism of the transfer cannot be reconstructed by sequence analysis. In spite of having no real clues to the mechanism, most authors regard them introgressed sequences without considering other possible transfer mechanisms (e.g., Liti et al., 2006; Muller and McCusker, 2009; Louis, 2011; Dunn et al., 2013; Boynton and Greig, 2014; Marsit and Dequin, 2015; Hou et al., 2016; Dujon and Louis, 2017; Guillamón and Barrio, 2017; Peris et al., 2017; Albertin et al., 2018). Hittinger et al. (2015) assumed that *S. cerevisiae* gains genes from other *Saccharomyces* species by introgression whereas from non-*Saccharomyces* species by horizontal gene transfer (HTG). This distinction was used in other works as well (e.g., Legras et al., 2018). Other authors use the latter term also for gene transfer between *Saccharomyces* species (Marinoni et al., 1999). Automatic attribution of all gene flow events within the genus to introgression appears to be a disputable oversimplification of a complex issue. By definition, introgression (or “introgressive hybridisation”) incorporates genes from one entity (species) into the gene pool of a second, divergent entity (species) via hybridisation and backcrossing of the hybrid with the latter entity (Anderson and Hubricht, 1938). But the hybrids of the *Saccharomyces* species are sterile, either produce no spores (no gametes suitable for backcrossing with the parent) at all, or if they sporulate, the frequency of the viable, usually highly aneuploid spores is very low and these spores are frequently unable to mate (e.g., Gjermansen and Sigsgaard, 1981; Zambonelli et al., 1993; Hawthorne and Philippsen, 1994; Kishimoto, 1994; Hunter et al., 1996; Marinoni et al., 1999; Liti et al., 2006; Kunicka-Styczyńska and Rajkowska, 2011; Pfliegler et al., 2012). Several recent works pointed out the conflict between the hypothesized (exclusive) role of introgression in mixing of genetic information of *Saccharomyces* species and its low chance of occurrance due to the sterility of the hybrids (e.g., Morales and Dujon, 2012; Marsit and Dequin, 2015; Dujon and Louis, 2017). Perhaps the more general term “genetic admixture” would be more accurate than “introgression” when the mechanism of the gene transfer is not known.

The alternative of introgressive backcrosses is the gradual genome reduction and chimerisation in meiotic and/or mitotic divisions, which will be referred to as GARMe and GARMi in the forthcoming sections. GARMe (Genome Autoreduction in Meiosis) generates chimeric genomes in series of successive meiotic divisions after the breakdown of the sterility barrier upon tetraploidisation (Karanyicz et al., 2017), whereas GARMi (Genome Autoreduction in Mitosis) is its counterpart operating during vegetative (mitotic) propagation of the hybrid cells. Both mechanisms are described in the forthcoming sections. Neither involves introgressive backcrosses and thus it is misleading to call their outcomes introgressions. Occasionally, the latter process is also called introgression (e.g., Dunn et al., 2013) despite the fact that it does not involve sexual interactions.

### Fertility – Sterility

A strain is fertile if it produces viable meiospores (ascospores) that can act as gametes capable of fusion (conjugation) with other meiospores. As the *Saccharomyces* meiospores can propagate vegetatively and produce clones of cells (spore clones), the ability to mate as a criterion of fertility applies also to these cells (“propagating gametes”). As discussed in the previous paragraph, the allodiploid interspecies hybrid is sterile because its cells neither produce viable spores (spore viability is usually much lower than 1%; e.g., Naumov et al., 2000; Delneri et al., 2003) nor mate with other cells. The allotetraploid hybrid is different because its cells are defective only in mating. The allotetraploid cells can sporulate and produce viable spores (e.g., Banno and Kaneko, 1989). However, the spores (spore clones) are usually sterile because they are usually allodiploid like the allodiploid hybrids (e.g., Pfliegler et al., 2012; Karanyicz et al., 2017) (for a detailed description, see the section on the second sterility barrier). Allotetraploid meiosis produces also alloaneuploid spores which can be sterile or fertile, depending on which chromosomes are missing (Antunovics et al., 2005; Pfliegler et al., 2012). If nullisomic for one of the *MAT*-carrying parental chromosomes, the alloaneuploids can mate and form alloaneuploid zygotes capable of sporulation (see section on GARMe). Several studies reported on interspecies *Saccharomyces* hybrids producing viable spores but often without providing information on the frequency of such spores, and/or on the ploidy of the hybrids and their spores (e.g., Greig et al., 2002b; Lee et al., 2008; Chou et al., 2010; Verspohl et al., 2018).

An important condition for viable spore production is homothallism, allowing the mating type switch in haploid/alloaneuploid cells after almost every division. Therefore, progeny of interspecies hybrids between different *Saccharomyces* species is able to establish fertile lines after sporulation and self-fertilization, provided they lose *MAT* heterozygosity (see section on the break-down of sterility barrier). In case of different biological species spores of allodiploid hybrids are rarely viable and only about 0.1–1% germinate and proliferate into visible colonies, from which many produce immortal lines (e.g., Naumov et al., 2000; Delneri et al., 2003 and many others).

### Generation Terminology

In the Mendelian terminology the pairing (mating) individuals constitute the P (parental) generation, their descendants (hybrids) are the first filial generation (F1), and the descendants of the pairing (or self-pollinating) F1 individuals are the F2 generation. For pairing, the sexually propagating animals and plants produce gametes by meiosis which then either fuse with other gametes to form zygotes (fertilization) or die. In contrast to animals and plants, the products of the yeast meiosis (meiospores, ascospores, basidiospores) do not die if they cannot mate. Instead, they germinate and form clones of vegetatively propagating cells. For these clones of “propagating gametes” there is no Mendelian term. In this review, they will be referred to as F1 spore clones. If the F1 spore is fertile and homothallic, the cells of its vegetative clone can switch mating types and then can mate with other cells of the same clone (selfing, autofertilisation) (Pfliegler et al., 2012). The resulting zygotes will propagate vegetatively, and can be considered F2 generation (equivalents of the F2 generation of self-pollinating plants). Their spores (gametes) are the F2 spores and the cells produced by the F2 spores are the F2 spore clones. If the F2 spore clone is homothallic, its cells can conjugate with each other and produce the F3 generation. This nomenclature will be used in this review. It may differ from those used in certain other works.

## The Complexity of (Allodiploid) Hybrid Sterility

Strains of the natural (“clean” or “single-genome-based”) *Saccharomyces* species are usually homothallic (for a review, see Rainieri et al., 2003). Their genomes are diploid or aneuploid and heterozygous for the mating-type alleles. *MATa/MATalpha* heterozygosity makes the cells unable to conjugate by repressing the mating program. However, it allows meiosis and sporulation in response to proper external signals (e.g., starvation). Meiosis produces haploid meiospores that have only one *MAT* locus, either *MATa* or *MATalpha*. The loss of *MAT* heterozygosity blocks the meiotic program and concomitantly abolishes the block of the mating program. So the spores can mate with other mating-competent conspecific spores and thus can act as gametes to form autodiploid zygotes. The zygotes then produce mitotically propagating autodiploid cells, equivalents of the somatic cells of higher eukaryotes. Having a *MATa/MATalpha* genotype, these cells are also sporogenic and can produce viable gametes (ascospores). For a review of the genetic determination of sexual processes of *Saccharomyces* strains, see Herskowitz (1988).

Mating can take place also between non-conspecific *Saccharomyces* spores. The resulting allodiploid zygotes are viable and produce vegetative cells much like the autodiploid zygotes. But in contrast to those, the allodiploid cells cannot sporulate or if they do so, their spores are very rarely viable (e.g., Gjermansen and Sigsgaard, 1981; Zambonelli et al., 1993; Hawthorne and Philippsen, 1994; Kishimoto, 1994; Hunter et al., 1996; Marinoni et al., 1999; Liti et al., 2006; Kunicka-Styczyńska and Rajkowska, 2011; Pfliegler et al., 2012). This phenomenon called hybrid sterility or hybrid incompatibility plays an essential role in the biological isolation of the *Saccharomyces* species because it impairs the competitiveness of the hybrids under unfavorable natural conditions (for explanation, see section the bad and good sides of sterility) and prevents the recombination of their gene pools.

What is the genetic basis of hybrid sterility in *Saccharomyces*?

Four major hybrid sterility mechanisms have been proposed:

### Interactions of Incompatibility Genes

Although nucleo-mitochondrial incompatibility is apparently the main reason for divergence of species, it is not involved substantially in the sterility of interspecific hybrids, because they contain both copies of incompatibility genes. Debilitated nucleo-mitochondrial communication has been reported only in haploid cybrids with reduced respiration or in non-respiring chimeras containing mitochondria from one partner as well as a set of original chromosomes, where one or two were replaced with their counterpart from second partner. However, these cells can mate and the respiration is rescued (Lee et al., 2008; Chou et al., 2010; Špírek et al., 2014). In principle, other forms of incompatibility with adverse effect on spore formation are also conceivable. For example, the spores could be killed by the interactions of recessive or dominant chromosomal incompatibility (speciation) genes. The aforementioned sporulation proficiency of the allotetraploid hybrids rules out the possibility that dominant incompatibility genes cause the sterility of allodiploids (Greig et al., 2002a). Efforts to find recessive incompatibility genes were unsuccessful (Greig, 2007; Kao et al., 2010). However, a recent computer simulation raised the possibility that two- and multilocus incompatibilities with incomplete penetrance could operate in *S. cerevisiae* × *S. paradoxus* hybrids (Li et al., 2013). Such interactions might also affect sporulation but incomplete penetrance is likely to cause only slight reduction of spore viability.

### Chromosome Rearrangement

Many of the spores from yeast hybrids are unviable, because they do not contain a complete genome’s worth of genes. In the case of single chromosomal translocation, 50% of the gametes will lack part of one of the translocated chromosome and die (Delneri et al., 2003).

### Misexpression of Meiotic Genes

Swain Lenz et al. (2014) found that the meiotic gene expression program proceeds more rapidly in *S. cerevisiae* × *S. paradoxus* hybrids than in the parents, and the change in the timing results in a heterochronic pattern of misexpression during midmeiosis. The authors hypothesized that the temporal changes might compromise the efficiency of meiosis. In a different study the fast evolution of the meiosis-related genes was hypothesized to account for hybrid sterility but the hypothesis was not tested experimentally (Xu and He, 2011). These models are also difficult to reconcile with the high spore viability of allotetraploids.

### Aberrant Chromosomal Behavior in Meiosis and Antirecombination

Numerous early studies have shown that the sterility of the hybrids of different plant and animal species is due to inadequate or deficient chromosome pairing during meiosis (e.g., Walters, 1958; John and Weissman, 1977; Gangadevi et al., 1985). Similar pairing aberrations were later observed also in hybrids of *Saccharomyces* species (Lorenz et al., 2002), shown to hamper chromosome segregation (Hawthorne and Philippsen, 1994; Sebastiani et al., 2002; Boynton and Greig, 2014) and implicated in hybrid sterility (e.g., Ryu et al., 1998; Delneri et al., 2003).

Chromosome alignment requires DNA strand exchange between similar segments of the pairing chromosomes (as part of recombination) (Sansam and Pezza, 2015). If the sequence similarity is low or restricted to shorter segments (e.g., between homeologous chromosomes of the subgenomes in the alloploid hybrid), there are fewer opportunities for strand exchange and thus the chromosomes cannot be properly aligned. Because of the sequence differences, strand exchange creates mainly heteroduplexes with mismatched nucleotides. Mismatches are targets for the mismatch repair machinery. This repair process can eliminate heteroduplexes and thus impair the pairing of the homeologous chromosomes (antirecombinational effect). The improvement in spore viability observed by Hunter et al. (1996) after the attenuation of the mismatch repair in *S. cerevisiae* and *S. paradoxus* hybrids might have been due to the elimination of fewer heteroduplexes (reduction of the antirecombinational effect). Until recently, mismatch repair was regarded a major player in the sterility barrier. However, recent results revealed that structural differences between the chromosomes alone are sufficient for reproductive isolation (Leducq et al., 2016). Besides, a recent work found that karyotype engineering can lead to reproductive isolation and eight chromosome–chromosome fusion events suffice to isolate a *S. cerevisiae* strain reproductively from other conspecific strains (Luo et al., 2018).

If the homeologous chromosomes differ in synteny, aberrant bivalents (syntenic segments having different positions in the homeologous chromosomes pair) (e.g., Karanyicz et al., 2017) and even partial multivalents (syntenic segments located on non-homeologous chromosome pairs) (Lorenz et al., 2002) can be formed. The meiotic spindle apparatus then cannot handle correctly the poorly and chaotically synapsed chromosomes and most probably either collapses (no sporulation) or forms nullisomic spores lacking essential genes and aneuploid spores with non-functional combinations of parental chromosomes (dead spores) (Hawthorne and Philippsen, 1994; Boynton et al., 2018). Thus, the probability of the formation of viable spores is very small and the proportion of haploids among them can be extremely low. Interestingly, in certain works, all viable spores were considered haploid without being subjected to any ploidy tests (Greig et al., 2002b; Lee et al., 2008; Chou et al., 2010). This, however, seems to be a disputable oversimplification in view of a recent estimation of alloaneuploid formation during allodiploid meiosis performed by the same authors (Boynton et al., 2018). Their model is difficult to reconcile with the results of Xu and He (2011), who found that the vast majority of the viable spores of their synthetic *S. cerevisiae* × *S. paradoxus* allodiploid had either *S. cerevisiae* or *S. paradoxus* haploid genomes. The reason for the discrepancy is not clear. Nevertheless, the production of spores with parental genomes would be a dead end on the road toward introgression and other modes of genome chimerisation.

## The Second (Yeast-Specific) Sterility Barrier Operating in Allotetraploids

**FIGURE 1:**
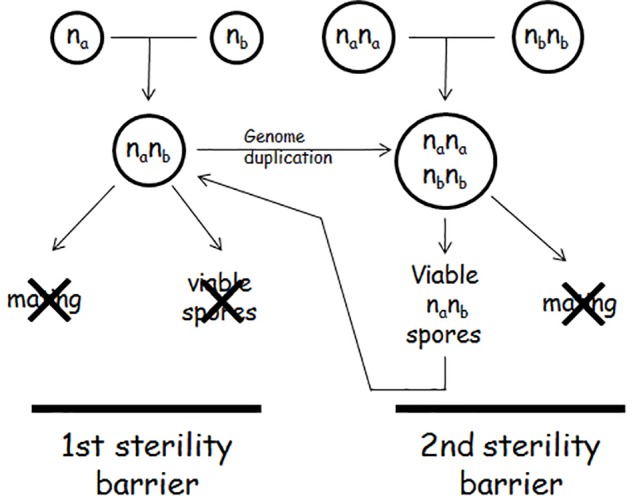
The double sterility barrier.

In plants, the sterile allodiploid hybrids become fertile when they duplicate their genomes because in the allotetraploid genomes each chromosome has a homologous partner to pair with, and thus a normal meiosis can take place. Upon genome duplication the hybrids produce viable and functional allodiploid gametes (for a review, see Ramsey and Schemske, 2002). A seemingly similar process takes place also in *Saccharomyces* interspecies hybrids but with a different outcome. If the hybridisation of two *Saccharomyces* species is followed by genome doubling or the mating cells were diploid (rare event), the hybrid becomes able to form viable spores (Cummings and Fogel, 1978; Banno and Kaneko, 1989; Marinoni et al., 1999; Johnston et al., 2000; Naumov et al., 2000; Greig et al., 2002a; Sebastiani et al., 2002; Antunovics et al., 2005; Pfliegler et al., 2012; Karanyicz et al., 2017). This observation led to the widespread view that genome duplication restores fertility in *Saccharomyces* interspecies hybrids as well. However, where investigated, these spores turned out to possess allodiploid chromosomal sets and thus represented a return to the ancestral sterile allodiploidy (Sebastiani et al., 2002; Pfliegler et al., 2012; Karanyicz et al., 2017) (Figure 1). Their sterility makes them different from the gametes of the allotetraploid plants which are fertile. Another difference from the plant allodiploid gametes is that the yeast allodiploid gametes (F1 spores) can propagate vegetatively and produce allodiploid F1 spore clones. The third difference is that the cells of these clones can sporulate. But like the spores of the allodiploid hybrids, the spores of the allodiploid F1 spore clones are dead. The inability of the F1 spore clones of the allotetraploid cells to mate and produce viable spores is the second sterility barrier in the biological isolation of the *Saccharomyces* species (Pfliegler et al., 2012). Thus, in contrast to the plant interspecies hybrids, genome duplication does not restore fertility in the *Saccharomyces* hybrids, it only produces non-functional gametes that can propagate vegetatively but cannot fertilize (conjugate).

## The Second Sterility Barrier Is Maintained by Autodiploidisation During the Allotetraploid Meiosis

The pairing of a chromosome with its homologous partner (autosyndesis) in the allotetraploid meiosis excludes its pairing with a chromosome from the other subgenome (allosyndesis) (Ramsey and Schemske, 2002; Moore, 2002). Thus, each diploid subgenome performs meiosis without interaction with the companion subgenome and then transmits its halved (haploid) sets of chromosomes into each of the four gametes. This mode of meiotic division is referred to as autodiploidisation of the allotetraploid genome in plant genetics (Hutchinson et al., 1983). The investigation of the viable spores produced by allotetraploid *Saccharomyces* hybrids revealed that the meiosis of the allotetraploid yeasts is also autodiploidised (Pfliegler et al., 2012; Karanyicz et al., 2017). Like the gametes of the allotetraploid plants, the spores of the allotetraploid yeasts are allodiploid with a single set of chromosomes in both subgenomes. Each subgenome contains a *MAT*-carrying chromosome but their *MAT* alleles are different because the diploid subgenomes of the allotetraploid were homozygous for different alleles: one was *MATa/MATa* the other was *MATalpha/MATalpha* (because hybridisation can take place between cells of opposite mating types and mating-type switching is then repressed by the *MAT* heterozygosity; Herskowitz, 1988). The simultaneous presence of both *MAT* alleles makes the spores (and their vegetative progeny) of the allotetraploid hybrid unable to mate but able to launch the meiotic program, although the spores produced are not viable (Pfliegler et al., 2012). This is exactly what characterizes the allodiploid hybrids. Thus, after genome duplication and a round of successful meiosis the hybrid returns to the sterile allodiploid state (Figure 1). This mode of meiosis and chromosomes segregation is very different from those characteristic of autotetraploids. In an autotetraploid *S. cerevisiae* cell any chromosome can freely pair with any of the other three homologous copies regardless of their origin. As a result, many allodiploid spores will be homozygous for the mating type (*MATa/MATa* or *MATalpha/MATalpha*) and capable of mating. Neither the heterozygous spores are sterile because their vegetative descendants produce viable haploid spores (e.g., Hilger, 1973; Al Safadi et al., 2010).

## Escape From the Biological Isolation: Breakdown of the (Second) Sterility Barrier by Loss of *Mat* Heterozygosity

**FIGURE 2:**
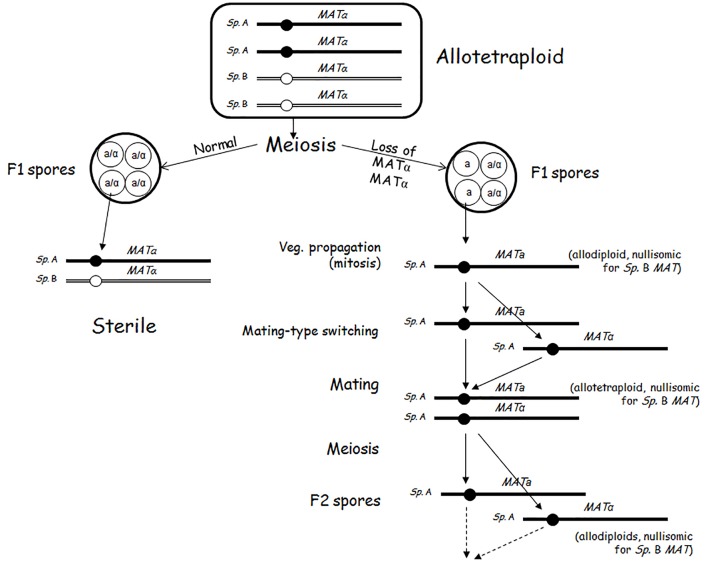
Breakdown of the sterility barrier by malsegregation of the *MAT*-carrying chromosomes of one subgenome in allotetraploid meiosis.

The allotetraploid meiosis turned out to be prone to errors at the distribution of the chromosomes to the spores. It was noticed that certain asci of certain allotetraploids contained two types of spores, usually in 2:2 proportion. Two spores were sterile, and two spores formed clones of cells producing viable spores (Antunovics et al., 2005; Pfliegler et al., 2012; Karanyicz et al., 2017). The latter type lacked the *MAT*-carrying chromosome in one of the subgenomes. This chromosome (designated III in *S. cerevisiae* and III, 3 or 2 in other species, depending on the numbering system used) is known to be the least stable chromosome even in autoploid *S. cerevisiae* strains (Kumaran et al., 2013). Having only one *MAT* allele (being hemizygous at the *MAT* locus), the cells of these clones can (in contrast to the clones of the *MATa/MATalpha* allodiploid spores) switch their mating types and then conjugate with each other to form uniparentally disomic allotetraploid zygotes. This can take place because the loss of mating-type heterozygosity reactivates both the mating-type switching machinery and the mating program (Herskowitz, 1988). The intraclonal conjugation (selfing, autofertilisation) converts the F1 spore clone to an F2 population of cells having allotetraploid genomes nullisomic for the lost *MAT*-carrying chromosome of one subgenome and disomic for the *MAT*-carrying chromosome of the other subgenome. In these cells autosyndetic (homologous) chromosome pairing can take place, and thus the meiosis produces fertile alloaneuploid F2 spores. The cells of the F2 spore clones then form a fertile F3 generation and so on. These observations demonstrate that the loss of *MAT* heterozygosity by the loss of the *MAT*-carrying chromosomes in one of the subgenomes abolishes the sterility of the alloploid hybrid (Figure 2). The conjugation-proficient spore clones of the tetraploid *S. cerevisiae* × *S eubayanus* hybrids used by Krogerus et al. (2017b) for hybridisation with a *S. cerevisiae* strain might have been this sort of fertile alloaneuploids. The cost of the breakdown of the sterility barrier is the destabilization of the hybrid genome. The loss of the *MAT*-carrying chromosome converts the euploid genome into aneuploid after which additional chromosomes can be lost in rapid succession (see next section).

In principle, conjugation-proficient spores can occur also among the few viable aneuploid spores produced by allodiploid hybrids provided they have only one *MAT*-carrying chromosome. The fertile “haploid” spores described in Greig et al. (2002b) and in Lee et al. (2008) could have been such segregants but because their ploidy was not examined, the possibility cannot be excluded that they were allodiploid spores nullisomic for one parental *MAT*-carrying chromosome produced by cells of duplicated (allotetraploid) genomes.

## Chimerisation of the Hybrid Genome in Meiotic Divisions: Introgression Versus Garme

The fertile alloaneuploid spores produced after the loss of *MAT* heterozygosity can conjugate with any fertile gamete of any other strain, also with the spores of one or the other parental species which opens the way for introgression. The caveat is that the products of these backcrosses will have low fertility due to their unbalanced sets of chromosomes and *MAT* heterozygosity. In an early work Sebastiani et al. (2002) observed conjugation between a few F1 spore clones of allotetraploids and the parental strains, but the hybrids obtained from the backcross produced mainly dead spores. de Barros Lopes et al. (2002) raised the possibility that introgression might take place by serial matings of the vegetative cells of the sterile hybrid with cells of one of the parental species [by the process called rare mating (Gunge and Nakatomi, 1972)]. Lee et al. (2008) managed to backcross the vegetative and autodiploidised (via self-fertilization) descendants of the spores of a *S. cerevisiae* × *S bayanus* (*uvarum*?) hybrid to the *S. cerevisiae* parent several times successively. Because of the very concise description of certain parts of the experimental procedure, it is impossible to resolve the inconsistency between the assumable *MATa/MATalpha* genotype (mating program repressed) of the cells and their ability to mate. It is conceivable that the spore clones contained conjugation-proficient segregants that had lost one of the parental *MAT*-carrying chromosomes. Nevertheless, the transfer of genetic information between *Saccharomyces* species via introgressive backcrosses has not been verified yet experimentally.

**FIGURE 3:**
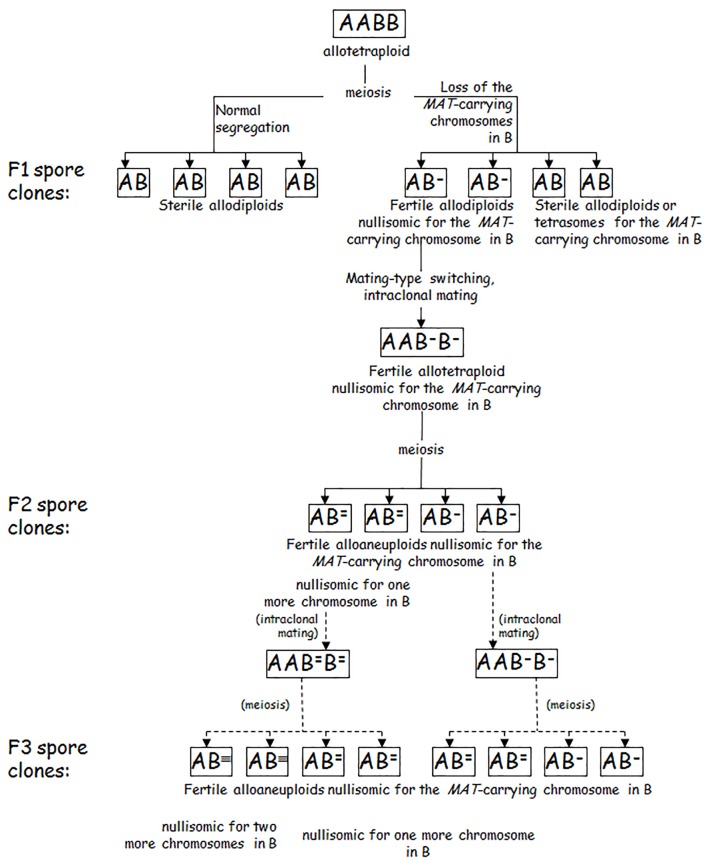
Genome autoreduction in meiosis (GARMe). A and B: parental genomes. The superscript hyphens represent lost chromosomes. Although all hyphens are shown over B, chromosomes can be lost from either subgenome. Chimeric genomes are produced when chromosomes are lost from both subgenomes, and allosyndetic interactions (recombination) take place between the subgenomes during meiosis.

A recently described alternative process leading to chimerised genomes is GARMe (Genome Autoreduction in Meiosis) (Karanyicz et al., 2017) (Figure 3). It is a progressive process starting with the breakdown of the sterility barrier by the malsegregation of the *MAT*-carrying chromosomes during the allotetraploid meiosis. The loss of a pair of *MAT*-carrying chromosomes destabilizes the genome and additional pairs of autosyndetically paired chromosomes can malsegregate in the meiotic divisions of the consecutive filial generations of autofertilised spore clones. The genome gradually becomes smaller at each meiotic division, with one partner’s chromosomes being lost preferentially. Occasionally, recombination between chromosomes of the subgenomes can also take place (Antunovics et al., 2005; Karanyicz et al., 2017; Krogerus et al., 2017b), most probably due to allosyndetic interactions between similar segments of homeologous chromosomes (Lorenz et al., 2002). During GARMe, the hybrid genome is gradually transformed into various genomic chimeras comprising predominantly chromosomes of one species but containing also mosaics from the genome of the other species (Antunovics et al., 2005; Pfliegler et al., 2012, 2014; Lopandic et al., 2016; Karanyicz et al., 2017). The process gradually leads to chimeric genomes similar to those found in “natural hybrids” (in fact chimeras) that have only a few genes from the donor genome (see above). The differences between the hybrids created by hybridizing conjugation-proficient spore clones of an allotetraploid *S. cerevisiae* × *S. eubayanus* hybrid and a *S. cerevisiae* strain (Krogerus et al., 2017b) might be attributed to different chromosome losses in the spores.

## Alteration of the Hybrid Genome During Vegetative (Mitotic) Propagation (Hybrid Evolution, Garmi)

Vegetatively propagating diploid *S. cerevisiae* cells can lose chromosomes by rare spontaneous chromosome non-disjunction during mitosis. The event generates two aneuploid daughter cells one of which is monosomic, the other being trisomic (Parry and Zimmerman, 1976). Aneuploidy destabilizes the genome and thus initiates further karyotype changes (e.g., Parry and Cox, 1970; Zhu et al., 2012). As predicted in the model proposed a decade ago (Sipiczki, 2008), malsegregation leading to aneuploidy can occur also in vegetatively propagating populations of allodiploid hybrid cells. Recent studies confirmed the prediction. It was found that alloploid *Saccharomyces* hybrids can lose chromosomes during vegetative propagation, which can also be accompanied by other forms of structural rearrangement in the genome (e.g., Kunicka-Styczyńska and Rajkowska, 2011; Piotrowski et al., 2012; Dunn et al., 2013; Bellon et al., 2013, 2015, 2018; Pérez-Través et al., 2014b; Mertens et al., 2015; Lopandic et al., 2016; Krogerus et al., 2017b; Peris et al., 2017). Such derivatives (frequently called “evolved hybrids”) constitute minor components of the population of the hybrid cells but can become relatively more abundant if the hybrid culture is cultivated under enriching conditions favoring their propagation for longer periods of time (for a recent review, see Lopandic, 2018). Various “enriching” or selective conditions have been applied to obtain strains with specific properties such as anaerobiosis (Kunicka-Styczyńska and Rajkowska, 2011), high temperature and alcohol content (Piotrowski et al., 2012), ammonium limitation (Dunn et al., 2013), glucose limitation, phosphate limitation and sulfate limitation (Smukowski Heil et al., 2017) sulfate limitation (Sanchez et al., 2017), grape must fermentation (Pérez-Través et al., 2012, 2014b; Bellon et al., 2013, 2018; Origone et al., 2018), fermentation at high sugar concentrations (Lopandic et al., 2016), cultivation in lager beer medium (Mertens et al., 2015), in xylose fermentation medium (Peris et al., 2017), etc.

The most frequently detected genome modifications were losses of chromosomes and changes of the genome size. An extreme example is the loss of the almost entire *S. uvarum* subgenome during culturing of the *S. cerevisiae* x *S. uvarum* hybrid at increased temperature (31–46.5°C) (Piotrowski et al., 2012). Preferential alterations and reduction of one subgenome was reported several times. For example Bellon et al. (2018) also detected chromosomal loss and much more rearrangements in the *S. uvarum* subgenome than in the *S. cerevisiae* subgenome during must fermentation. Lopandic et al. (2016) observed chromosome loss in the *S. uvarum* subgenome of two F1 spore clones of a *S. cerevisiae x S. uvarum* allotetraploid and in the *S. kudriavzevii* subgenome of F1 spore clones of a *S. cerevisiae x S. kudriavzevii* allotetraploid hybrid during experimental wine fermentation.

In addition to aneuploidisation of the genome, also size changes of certain chromosomes were observed (e.g., Kunicka-Styczyńska and Rajkowska, 2011; Lopandic et al., 2016; Bellon et al., 2018) which indicates that various forms of recombination (e.g., exchange of chromosomal arms, translocation) can occur too. Other experiments demonstrated that recombination can take place also at the level of individual genes. Dunn et al. (2013) detected frequent recombination events between the *MEP2* genes of the partner genomes in vegetatively propagating *S. cerevisiae* × *S. uvarum* hybrids. The observed genomic changes and interactions between the subgenomes in vegetatively propagating hybrids strongly suggest that the hybrid genome can be reduced and chimerised not only by GARMe but also during vegetative propagation without introgressive backcrosses. To the analogy of GARMe, this process can be called GARMi (Genome Autoreduction in Mitosis).

Different selective conditions can enrich derivatives of different phenotypes from the same hybrid (e.g., Piotrowski et al., 2012). When there is no selective pressure, the culture can segregate into subpopulations differing in genome size and chromosomal constitution even in the same culturing conditions (e.g., Mertens et al., 2015). The phenotypic diversity found in a recent study among hybrids obtained by hybridisation of the same pair of isogenic strains (Verspohl et al., 2018) might have been the consequence of different spontaneous genome rearrangements in the individual hybrid lines during their maintenance in non-selective culturing media.

The overgrowth of the unchanged hybrid cells by a segregant is a sort of adaptive evolution at population level because the cells of the segregant (evolved hybrid) are more fit than the original hybrid under the applied culturing conditions. The winner then remains stable, thus the process can also be regarded genome stabilization (e.g., Piotrowski et al., 2012; Pérez-Través et al., 2014b; Krogerus et al., 2017b; Bellon et al., 2018) but cultivation of the “stabilized” strain under different conditions would show whether its genome is stable indeed.

The aneuploid spore clones produced after the breakdown of the sterility barrier also change during vegetative propagation. Lopandic et al. (2016) found extensive segregation in F1 spore clones of *S. cerevisiae* × *S. uvarum* and *S. cerevisiae* × *S. kudriavzevii* hybrids, whereas Krogerus et al. (2017b) observed less drastic changes in *S. cerevisiae* × *S. eubayanus* hybrids. In both cases the non-*cerevisiae* subgenome was less stable.

When interpreting the changes of the hybrid genome during vegetative propagation, it has to be borne in mind that occasional meiotic divisions may also contribute to the process despite the adverse cultivation conditions for sporulation. Mortimer et al. (1994) observed sporulation in certain wine yeast strains even in sugar-rich media and Sebastiani et al. (2002) found that *S. cerevisiae* × *S. uvarum* hybrids could form asci on YPD (yeast-peptone-glucose medium).

In principle, GARMi can also break down the sterility barrier, if the hybrid cell loses the *MAT*-carrying chromosome in one of the subgenomes (loss of *MAT* heterozygosity). Kunicka-Styczyńska and Rajkowska (2011) found fertile segregants in cultures of sterile *S. cerevisiae* × *S. uvarum* hybrids after 50–80 generations of mitotically dividing cells.

## Why Is the Postzygotic Genome Reduction Asymmetric?

As shown above, one of the subgenomes usually undergoes a faster and more extensive reduction both in the meiotic (GARMe) and in the mitotic (GARMi) divisions. The less stable subgenome in meiosis was that of S. *uvarum* in the *S. cerevisiae* × *S. uvarum* hybrids (Antunovics et al., 2005; Pfliegler et al., 2012; Piotrowski et al., 2012), *S. eubayanus* in the *S. cerevisiae* × *S. eubayanus* hybrids (Krogerus et al., 2017b), *S. kudriavzevii* in the *S. cerevisiae* × *S. kudriavzevii* (Pfliegler et al., 2014; Lopandic et al., 2016) and *S. kudriavzevii* × *S. uvarum* hybrids (Karanyicz et al., 2017). The mechanisms underlying the unequal reduction of the subgenomes (concerted loss of chromosomes in one of the subgenomes) are unknown but a hypothetical model was recently proposed to explain the asymmetry (Karanyicz et al., 2017). The model is based on the assumption that the loss of a chromosome is unlikely to affect physically the segregation of other chromosomes. More plausibly, it creates a situation in which the subsequent loss of certain chromosomes of the other subgenome would have deleterious effects. For example, when the fertility is restored by the loss of the *MAT*-carrying chromosome of one of the subgenomes, all genes located on that chromosome are lost. The absence of these genes has no discernible effect on viability because their orthologs in the companion subgenome can substitute them. Most of the orthologs are in the *MAT*-carrying chromosome of the partner subgenome but some of them are on different, non-homeologous chromosomes [e.g., as shown in *S. kudriavzevii* × *S. uvarum* hybrids (Karanyicz et al., 2017)]. If one of these non-homeologous chromosomes is then lost during the next meiosis, the orthologues located on it will be eliminated from the genome. If any of them performs an essential function in the life of the cell, the spore lacking this chromosome will die. The dead spores in the F1 and F2 tetrads (Pfliegler et al., 2012; Karanyicz et al., 2017) can be the products of lethal combinations of chromosome losses in the subgenomes. The loss of another chromosome from the subgenome from which the *MAT*-carrying chromosome was lost is not entailed with similar risk but can render additional chromosomes of the partner subgenome essential for viability. Thus, the spores that lose chromosomes from the same subgenome have better chances to remain viable. Because of the differences in gene contents even between chromosomes considered homeologous, random losses of chromosomes are likely to eliminate essential genes and thus lead to non-functional genomes. Retaining mainly the chromosomes of one subgenome and reducing preferentially the other subgenome also prevents eventual clashes of the different regulatory networks of the hybridized species (e.g., Tirosh et al., 2010; Schraiber et al., 2013; Metzger et al., 2017). It is pertinent to mention here that structural and regulatory incompatibilities have been assumed to shape the highly diverse alloaneuploid genomes in the *S. pastorianus* lager strains (for a review, see Krogerus et al., 2017a). The question remains as to why the same subgenome loses its *MAT* chromosomes in different hybrids of the same species pair. A similar model can be proposed for the postzygotic genome evolution during the vegetative propagation of the hybrid cells.

## Biotechnological Aspects

The combination of the gene pools of two or even more *Saccharomyces* species by natural mating of cells and the postzygotic chimerisation of the hybrid genomes by natural meiotic and mitotic segregation have great potentials in breeding of novel production strains for food industry. These processes generate a great diversity of new phenotypes without producing GMO strains with targeted genetic modifications. Both hybridisation and segregation are natural processes that take place in the nature without human intervention. The role of the human activity is to guide these processes in experimental conditions so that new phenotypes useful in fermentation and food industry can emerge.

**FIGURE 4:**
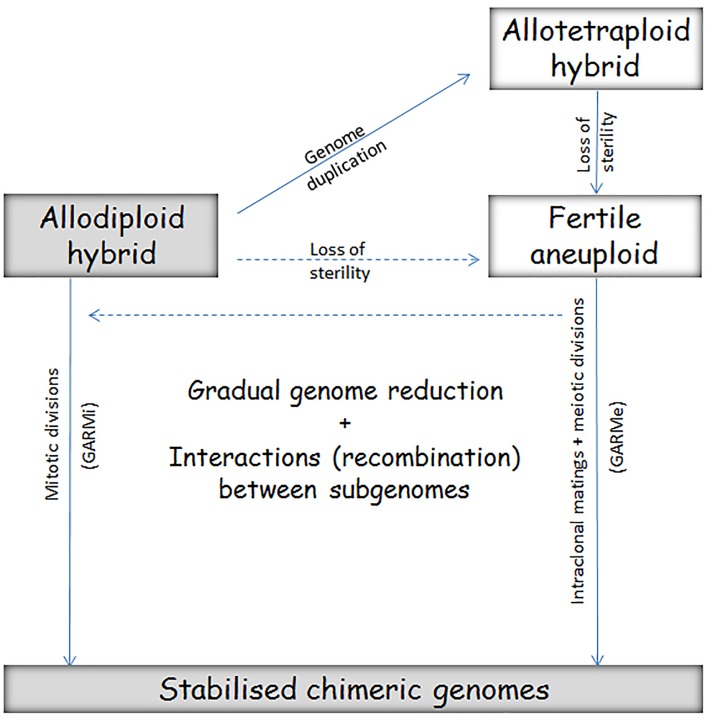
Two pathways of conversion of the interspecies hybrid into chimeric strains through genetically unstable intermediates. The conversion by serial biased mitotic segregation and recombination of chromosomes during vegetative propagation (GARMi) is exploited in the breeding strategy called adaptive evolution. As the outcomes are chimeric strains (“evolved hybrids”) that are genetically stable under the selective conditions that preferentially promoted their growth, the process is also called genetic stabilization of the hybrid. The alternative pathway leads to similar chimeric strains through biased segregation and recombination in a series of successive meiotic divisions (GARMe). The key events in this process are genome duplication, breakdown of sterility and autofertilisation (self-conjugation/mating) in the spore clones. Switching from one pathway to the other is possible (broken arrows). The fertile spores produced during GARMe can mate also with other fertile spores and cells of parental strains (a possibility for introgression). The allodiploid hybrids and the final chimeric strains are more suitable for biotechnological applications than the forms being in intermediary stages because the latter are less stable and may change unpredictably during propagation in the technology.

The hybrids differ in phenotype from the parental species, but the differences can be both favorable and unfavorable. During the chimerisation process the nascent hybrid can get rid of “bad” genes or alleles, retain and combine their “good” counterparts and create new regulatory networks allowing better adaptation to technological environments. From the technological point of view, the allodiploid hybrids and the chimerised end products of the postzygotic genome evolution are better suited to the technological demands because their genomes are more stable than those of the intermediate forms (Figure 4). Examples of successful applications of these processes are shown in Tables 1–3, and certain major conclusions are discussed in the following sections.

**Table 1:** Examples of interspecies hybridisation: phenotypes of the hybrid strains.

Species combination	New phenotype	Reference
*S. cerevisiae* × *S. arboricola*	Maltotriose fermentation, increased production of higher alcohols, esters and other aroma compounds in beer	Nikulin et al., 2018
*S. cerevisiae* ale × *S. bayanus*	Improvement of the fermentation performance of the ale yeast at low temperatures in wort fermentation	Sato et al., 2002
*S. cerevisiae* × *S. carlsbergensis* (lager) *S. cerevisiae* (ale) × *S. carlsbergensis* (lager)	Improved growth at higher temperatures and improved resistance against high osmolality or high ethanol concentrations; improved fermentation rates at 18–25°C	Garcia Sanchez et al., 2012
*S. cerevisiae* × (natural *S. cerevisiae* × *S. kudriavzevii* chimeric strain)	Combination of low H_2_S production with improved ester production	Bizaj et al., 2012
*S. cerevisiae* × *S. eubayanus*	Combined phenotypic traits of the *S. cerevisiae* parent (growth at 35°C, utilization of maltotriose) and the *S. eubayanus* parent (efficient growth at low temperature) in synthetic wort	Hebly et al., 2015
	Improved fermentation power at low temperatures in cider and wine; no sulfurous off flavors are produced	Magalhães et al., 2017a,b
	Reduced 4-vinyl guaiacol formation	Diderich et al., 2018
	Maltotriose fermentation, increased production of higher alcohols, esters and other aroma compounds in beer	Nikulin et al., 2018
*S. cerevisiae* ale × *S. eubayanus*	Increased fermentation rates and flocculation, increased maltose and maltotriose utilization rates, higher concentrations of esters	Krogerus et al., 2015, 2016, 2017b
Engineered xylose-consuming *S. cerevisiae* × *S. eubayanus*	Intermediate growth rate, xylose consumption	Peris et al., 2017
*S. cerevisiae* × *S. kudriavzevii*	Decreased production of acetic acid, 3-methylbutanoic acid and ethyl acetate; increased production of ethyl hexanoate, ethyl butanoate and ethyl propanoate, 2-methylpropyl acetate, hexanoic acid and butanol	Bellon et al., 2011
	Higher fermentation rate, higher ethanol concentration, less residual sugar in wine compared to parental strains	Lopandic et al., 2016; Gangl et al., 2017
Engineered xylose-consuming *S. cerevisiae* × *S. kudriavzevii*	Intermediate growth rate	Peris et al., 2017
*S. cerevisiae* × *S. mikatae*	Concentrations of volatile metabolites different from those produced by *S. cerevisiae* in wine; increased amount of glycerol and low acetic-acid concentration	Bellon et al., 2013
	Maltotriose fermentation, increased production of higher alcohols, esters and other aroma compounds in beer	Nikulin et al., 2018
Engineered xylose-consuming *S. cerevisiae* × *S. mikatae*	Intermediate growth rate, xylose consumption	Peris et al., 2017
*S. cerevisiae* × *S. paradoxus*	Decreased production of acetic acid, 3-methylbutanoic acid and ethyl acetate; increased production of ethyl hexanoate, ethyl butanoate and ethyl propanoate, hexanoic acid and butanol	Bellon et al., 2011
	Better growth of certain hybrids in media supplemented with various amounts of ethanol, acetic acid, glucose, hydrogen peroxide, lithium acetate, sodium chloride and cycloheximide	Stelkens et al., 2014
	Hybrids grew better than their parents in direct competition.	Bernardes et al., 2017
*S. cerevisiae x S. uvarum*	Wider temperature range of high growth rate and fermentation velocity; intermediate production of malic acid, acetic acid, glycerol and certain flavor components	Kishimoto, 1994
	Increased fermentative vigor and wider temperature range in wine fermentation	Zambonelli et al., 1997; Rainieri et al., 1998
	Low acidity due to increased malic acid degradation, high glycerol production, wider temperature range in wine fermentation	Rainieri et al., 1999
	Increased polyphenol content in wine	Caridi et al., 2002
	Release of high amounts of volatile thiols produced from the *S*-cysteine conjugate precursor without producing excessive amounts of β-phenylethyl alcohol during wine fermentation	Masneuf et al., 2002
	Reduced ethanol, acidity, malic acid, lactic acid acetic acid production, increased free SO2	Rajkowska et al., 2005; Kunicka-Styczyńska and Rajkowska, 2011, 2012
	Lower pH sensitivity	Serra et al., 2005
	Wider optimum temperature of fermentation in wine fermentation	Solieri et al., 2005
	Improved flocculation and wider temperature range in sparkling wine fermentation	Coloretti et al., 2006
	Low level of volatile acidity, high level of glycerol, malic and succinic acid production, improved sensory quality in wine	Restuccia et al., 2011
	Higher growth and fermentation rate, higher ethanol and glycerol concentrations, lower concentrations of volatile acids, less residual sugar in wine compared to parental strains	Pfliegler et al., 2014; Lopandic et al., 2016; Gangl et al., 2017
	Low volatile acidity and novel aroma and flavor profiles in wines made from high-sugar and botrytized must	Bellon et al., 2015
	Increased ethyl-esters, less acetic acid, phenyl-2-ethanol and phenyl-2-ethanol acetate, with improved oenological performances and better homeostasis with respect to temperature in wine fermentation	da Silva et al., 2015
	Maltotriose fermentation, increased production of higher alcohols, esters and other aroma compounds in beer	Nikulin et al., 2018
	Broader temperature range; heterogeneous but mostly intermediate levels of stress sensitivity and production of ethanol and glycerol	Verspohl et al., 2018
Engineered xylose-consuming *S. cerevisiae* × *S. uvarum*	Improved growth rate and xylose consumption	Peris et al., 2017

**Table 2:** Examples of mitotic segregants of interspecies hybrids.

Species combination	Selective (enriching) condition	New phenotype	Reference
*S. cerevisiae* ale × *S. eubayanus S. cerevisiae* wine × *S. eubayanus*	Lager beer medium	Greater diversity of aroma compounds, increased ethanol production, broader temperature tolerance than that of the parental strains and the reference *S. pastorianus* strains	Mertens et al., 2015
*S. cerevisiae* × *S. kudriavzevii*	Wine fermentation	Fit for fermentation of synthetic must	Pérez-Través et al., 2014b
Engineered xylose-consuming *S. cerevisiae* × *S. kudriavzevii*	AFEX-pretreated corn stover hydrolysate medium	Improved xylose fermentation	Peris et al., 2017
Engineered xylose-consuming *S. cerevisiae* × *S. mikatae*	AFEX-pretreated corn stover hydrolysate medium	Improved xylose fermentation	Peris et al., 2017
*S. cerevisiae x S. uvarum*	Cultivation in laboratory medium under aerobic and anaerobic conditions	Acquired the ability to assimilate seven compounds ((2-keto-D-gluconate, adonitol, xylitol, inositol, sorbitol, *N*-acetyl-D-glucosamine and lactose). Diverse production of ethanol, glycerol and acids compared to the original hybrids. Partial restoration of fertility	Rajkowska et al., 2005; Kunicka-Styczyńska and Rajkowska, 2011, 2012
	High temperature, high ethanol concentration	Increased thermotolerance and modified cell-wall composition compared to parents	Piotrowski et al., 2012
	Ammonium limitation	Better growth in nitrogen-poor environment	Dunn et al., 2013
	Glucose, phosphate, and sulfate limitation	Increased copy number and loss of heterozygosity of certain genes involved in the adaptation to the selective conditions applied	Smukowski Heil et al., 2017
	Sulfate limitation	Increase of copy numbers of sulfate transporter genes	Sanchez et al., 2017
	Wine fermentation	Increased glycerol, malic acid, isobutyl alcohol and 1-propanol level in wine. Growth in a wider temperature range	Origone et al., 2018
	Grape-juice fermentation	Low volatile acidity and novel aroma and flavor profiles in wines	Bellon et al., 2018
Engineered xylose-consuming *S. cerevisiae* × *S. uvarum*	AFEX-pretreated corn stover hydrolysate medium	Improved xylose fermentation	Peris et al., 2017

**Table 3:** Examples of meiotic segregants of interspecies hybrids.

Species combination	Meiotic product	New phenotype	Reference
*S. cerevisiae* × *S. kudriavzevii*	F1 spore clones	Highly heterogeneous phenotypes	Pfliegler et al., 2014; Lopandic et al., 2016; Gangl et al., 2017
*S. cerevisiae × S. uvarum*	Mixture of F1, F2 hybrids, haploid and aneuploidy spore clones	Diverse phenotypes depending on the genotypes of the randomly mating F1 spores	Piotrowski et al., 2012
	F1 spore clones	Higher fermentation and growth rate, higher ethanol and glycerol concentrations in wine compared to parental strains	Pfliegler et al., 2014; Lopandic et al., 2016; Gangl et al., 2017
(*S. cerevisiae* × *S. eubayanus*) F1 spore × *S. cerevisiae*	Hybrid of meiotic spore clone with a third strain	Increased fermentation rate and maltotriose consumption, high ethanol production in wort and high concentrations of esters in beer, no 4-vinyl guaiacol production	Krogerus et al., 2017b

### Hybridisation Generates Both Favorable and Unfavorable Phenotypes

Hybrids of different species usually show phenotypes, referred to as transgressive phenotypes that differ from those of their parents. Transgressive phenotypes can be products of additive effects of the orthologous parental genes, epistatic interactions and/or novel regulatory networks for biochemical processes not easily predicted from the properties of the parental strains. The transgressive phenotypes can be either positive or negative in terms of fitness or technological usefulness. While many of these phenotypes are of no obvious value for food industry, there are a number of documented cases where novel beneficial traits have appeared in the hybrids. These hybrids can outperform their parental strains in one or more properties of technological relevance. Such positive examples are listed in Table 1. The most frequently observed positive traits are faster fermentation rates in wider temperature ranges, more efficient sugar utilization, better stress tolerance, broader or better aroma profiles (increased complexity of the sensory properties), reduced production of acetic acid, SH_2_ and other compounds with adverse effects on the quality of the fermented products. Some of these traits confer a competitive fitness advantage to the hybrid cells in the fermentation environment. The positive transgression, the superior performance of the hybrid compared to either of its parent is frequently referred to as heterosis or hybrid vigor (e.g., Krogerus et al., 2017b). Negative transgressive phenotypes are rarely published but one must be aware of the risk of fitness reduction and the production of undesirable off-flavors (Steensels et al., 2014; Krogerus et al., 2016).

### Hybridisation of the Same Parents Can Result in Diverse Hybrids

Hybrids of a pair of strains frequently show phenotypic diversity (e.g., Kunicka-Styczyńska and Rajkowska, 2011; Piotrowski et al., 2012; Lopandic et al., 2016). This phenomenon can be attributed to intragenomic heterogeneity of the parental strains. Wine and beer strains are usually highly heterozygous, frequently also aneuploid, and thus produce spores with diverse combinations of alleles and copy numbers of chromosomes (e.g., Johnston et al., 2000; Sipiczki et al., 2001, 2004; Steensels et al., 2014). Spores (spore clones) of different genomes form genetically and phenotypically different hybrids. Another reason for the heterogeneity can be the inherent instability of the hybrid genomes. If the hybrids of the same pair of parents are cultured for longer periods of time in non-selective laboratory media, they can become different due to random mitotic or meiotic segregation events as discussed above. Hybrid diversity reduces the predictability of the outcome of the breeding program but extends the range of phenotypes from which the breeder can choose.

### The Good and Bad Sides of Sterility

The inability of the allodiploid hybrids to produce viable spores has two important consequences of practical relevance. On the one hand, it keeps the genome stabile by preventing meiotic segregation; on the other hand it reduces the survival chances of the hybrid under stress conditions. Since allodiploid meiosis is abortive, the hybrid genome can change only by mitotic segregation, which is a much slower process than meiotic segregation (see above). Genetic stability is particularly important in the brewing industry where a yeast culture is reused multiple times (for a review, see Gibson et al., 2017). In the brewing technology the negative side of hybrid sterility, the high mortality of hybrids under stress conditions does not cause problems because the environment does not change much. But in natural wine-making, the fermenting yeast populations are suddenly exposed to adverse conditions after the completion of fermentation and have to withstand multiple stresses until the next vintage season. Wine yeasts can survive these periods on the winery equipment (Rosini, 1984; Martini, 2003), in vineyard soil (Cordero-Bueso et al., 2011) or in mummified grape berries (Sipiczki, 2016), where the ability to produce ascospores greatly increases their chances of survival. Spores are not only gametes but also dormant resting cells resistant to many stress conditions deleterious to vegetative cells (Honigberg, 2016). The hybrids are easily selected out from the population because they have only vegetative cells that die under conditions which the spores of the natural strains withstand. The lack of viable spores is a severe disadvantage that may account (synergistically with other factors such as genetic instability) for the rare occurrence of true hybrids in the nature. In inoculated wine fermentation the sterility of the starter yeast strain is irrelevant.

### Postzygotic Genomic Changes Broaden the Phenotypic Diversity

The inherent instability of interspecific yeast hybrids can be exploited for obtaining segregants (evolved hybrids) of chimeric genomes that display phenotypes outside the range of variation observed in the parents and the hybrids. The changes of the hybrid genome during the vegetative propagation of the cells (GARMi) are most probably spontaneous random events that take place independently of the culturing conditions. However, by culturing the population in a specific medium (e.g., high sugar content, low concentration of nitrogen sources) or under specific conditions (e.g., low or high temperature), the segregant having the best suited phenotype gradually overgrows the unchanged hybrid cells and the other segregants in the population (adaptive evolution). Examples of successful application of this experimental approach are listed in Table 2. Meiotic division also generates segregants with valuable novel phenotypes, provided that the hybrid can produce viable spores. Allotetraploids form viable spores but because of the autodiploidisation of the meiosis (see above) most spores are allodiploid having complete parental chromosomal sets. These are unlikely to show much difference in phenotype from the “ancestral” hybrid. But the alloaneuploid spores produced during GARMe can have chimerised genomes that express favorable novel phenotypes. Table 3 shows examples of strain improvement by generating spore clones from alloploid hybrids. The diversity can be further broadened by crossing the fertile spore clones with each other or with cells of different strains (e.g., Piotrowski et al., 2012). The drawback of strain improvement by meiotic segregation of the hybrids is the high instability of the fertile spore clones. Attempts have been made to obtain stabilized derivatives of spore clones by propagating them under technological conditions (e.g., Lopandic et al., 2016).

## Author Contributions

The author confirms being the sole contributor of this work and has approved it for publication.

## Conflict of Interest Statement

The author declares that the research was conducted in the absence of any commercial or financial relationships that could be construed as a potential conflict of interest.
